# Non-Invasive Dry Eye Disease Detection Using Infrared Thermography Images: A Proof-of-Concept Study

**DOI:** 10.3390/diagnostics15162084

**Published:** 2025-08-20

**Authors:** Laily Azyan Ramlan, Wan Mimi Diyana Wan Zaki, Marizuana Mat Daud, Haliza Abdul Mutalib

**Affiliations:** 1 Department of Electrical, Electronic and Systems Engineering, Faculty of Engineering and Built Environment, Universiti Kebangsaan Malaysia, UKM Bangi 43600, Malaysia; p145208@siswa.ukm.edu.my; 2 Institute of Visual Informatics, Universiti Kebangsaan Malaysia, UKM Bangi 43600, Malaysia; marizuana.daud@ukm.edu.my; 3 Optometry and Vision Science, Faculty of Health Sciences, Universiti Kebangsaan Malaysia, Kampus Kuala Lumpur, Kuala Lumpur 50300, Malaysia; halizamutalib@ukm.edu.my

**Keywords:** Dry Eye Disease, infrared thermography, machine learning, statistical analysis

## Abstract

**Background/Objectives:** Dry Eye Disease (DED) significantly impacts quality of life due to the instability of the tear film and reduced tear production. The limited availability of eye care professionals, combined with traditional diagnostic methods that are invasive, non-portable, and time-consuming, results in delayed detection and hindered treatment. This proof-of-concept study aims to explore the feasibility of using smartphone-based infrared thermography (IRT) as a non-invasive, portable screening method for DED. **Methods:** This study included infrared thermography (IRT) images of 40 subjects (22 normal and 58 DED). Ocular surface temperature changes at three regions of interest (ROIs): nasal cornea, center cornea, and temporal cornea, were compared with Tear Film Break-up Time (TBUT) and Ocular Surface Disease Index (OSDI) scores. Statistical correlations and independent *t*-tests were performed, while machine learning (ML) models classified normal vs. DED eyes. **Results:** In these preliminary results, DED eyes exhibited a significantly faster cooling rate (*p* < 0.001). TBUT showed a negative correlation with OSDI (r = −0.802, *p* < 0.001) and positive correlations with cooling rates in the nasal cornea (r = 0.717, *p* < 0.001), center cornea (r = 0.764, *p* < 0.001), and temporal cornea (r = 0.669, *p* < 0.001) regions. Independent *t*-tests confirmed significant differences between normal and DED eyes across all parameters (*p* < 0.001). The Quadratic Support Vector Machine (SVM) achieved the highest accuracy among SVM models (90.54%), while the k-Nearest Neighbours (k-NN) model using Euclidean distance (k = 3) outperformed overall with 91.89% accuracy, demonstrating strong potential for DED classification. **Conclusions:** This study provides initial evidence supporting the use of smartphone-based infrared thermography (IRT) as a screening tool for DED. The promising classification performance highlights the potential of this approach, though further validation on larger and more diverse datasets is necessary to advance toward clinical application.

## 1. Introduction

Dry Eye Disease (DED) is a condition in which the eyes fail to produce adequate tears or maintain a stable tear film, both of which are essential for ocular health and comfort. DED is primarily characterized by an imbalance in tear film homeostasis, accompanied by ocular discomfort resulting from tear film instability, hyperosmolarity, inflammation, surface damage, and neurosensory dysfunction [1]. It is generally classified into two primary types: tear-deficient dry eye and evaporative dry eye, with further subclassifications based on various intrinsic and extrinsic etiological factors [2,3]. Notably, the National Eye Institute/Industry Workshop on Clinical Trials in Dry Eyes defines dry eye as “a disorder of the tear film due to tear deficiency or excessive tear evaporation,” highlighting these two mechanisms as the primary causes of the condition. The severity of dry eye symptoms varies among individuals and may include ocular discomfort, pain, fatigue, and visual disturbances, including blurry vision [4]. These symptoms can significantly impact daily life, as persistent discomfort and vision issues may interfere with activities like reading and driving. Additionally, the pain and irritation associated with dry eye can negatively affect mental well-being and overall quality of life. Beyond personal challenges, reduced visual clarity and discomfort may also hinder work productivity, ultimately influencing both individual success and broader economic outcomes.

Multiple clinical studies have highlighted the significant role of inflammation in the ocular surface, meibomian glands, lacrimal glands, autoimmune diseases, and systemic conditions, as well as age-related dysfunction, in the pathogenesis of DED [5,6,7]. Additional factors, including female gender, certain medications, corneal nerve sensitivity loss, reduced humidity, and increased exposure to wind, contribute to the dysfunction of the lacrimal functional unit (LFU), which is essential for tear film production and stability [8]. Given the multifactorial nature of DED and its impact on tear film stability, accurate diagnosis is essential for effective management. The TFOS DEWS II guidelines recommend subjective clinical tests such as the McMonnies Questionnaire or the Ocular Surface Disease Index (OSDI), followed by objective clinical tests like the Schirmer test and tear film breakup time (TBUT) [9]. However, these objective tests can be invasive, time-consuming, and often lack consistency, particularly in mild to moderate cases [10]. Given these limitations, there is an increasing need for a non-invasive, rapid, sensitive, and portable screening tool for more effective DED detection.

Infrared Thermography (IRT) is a non-invasive and portable technology designed to measure infrared radiation or surface temperature emitted by objects or body areas. In medical applications, IRT has been widely utilized to assess temperature variations across surfaces, organs, tissues, and cells, serving as an indicator of potential abnormalities [11]. Since its pioneering use in by [12] for measuring ocular surface temperature (OST) in dry eye patients, IRT has gained significant attention in the screening and research of DED due to its non-ionizing, safe, and contact-free nature. Numerous studies have since reported distinct OST patterns between normal and DED-affected eyes, despite variations in IRT device specifications [10,13,14,15]. The core objective across these studies remains the identification of reliable thermal characteristics that can effectively differentiate DED patients from healthy individuals.

The urgency to develop efficient DED screening methods is particularly pronounced in Malaysia, where research and data on DED prevalence are still limited. A study by [16] highlighted a concerning DED prevalence rate of 48.8% in Malaysia, which is well above global estimates of 7% to 34%. This further emphasizes the need for greater awareness, early detection, and effective management strategies to safeguard visual health and quality of life. This high variability in prevalence is influenced by inconsistent diagnostic criteria, differences in tear film assessments, demographic diversity, and lifestyle factors, with findings particularly relevant to populations such as those in Kuantan.

The availability of optometry professionals remains limited in Malaysia, which can impact efforts to improve ocular health services. According to the 2024 health indicators report, the country’s optometrist-to-population ratio stands at 1:12,729 [17], significantly below the internationally recommended ratio of 1:10,000 [18]. Traditional DED diagnostic methods, while accurate and clinically reliable, are often expensive and not widely available, particularly in rural or underserved areas. This lack of accessibility contributes to delayed diagnosis and treatment, increasing the risk of chronic ocular complications, including potential vision loss.

To address these growing challenges, especially in regions with limited access to specialized care, there is a clear demand for non-invasive, cost-effective, and portable screening solutions. Conventional diagnostic tools, despite their precision, are often impractical due to financial and logistical constraints. In this context, IRT, particularly when integrated with smartphone-based thermal cameras, presents a promising alternative for rapid and accessible DED detection. Furthermore, advancements in machine learning (ML) and deep learning (DL) have enhanced the ability to automatically extract and classify key thermal features that differentiate DED from normal eyes.

Therefore, this study introduces an innovative, non-invasive approach for the early screening of DED by integrating IRT with ML techniques. This preliminary study serves as proof-of-concept, aiming to explore the potential of this combined methodology for enabling early, efficient, and scalable screening. It provides a practical solution for enhancing DED management, particularly in settings with limited healthcare resources. The remainder of this paper is structured as follows: Section 2 describes the materials and methods, focusing on the use of IRT images for DED screening; Section 3 and Section 4 present the results and subsequent discussion; and Section 5 concludes the study with key findings and future perspectives. This work is part of the ASEAN IVO project focused on developing an integrated system for ocular disease detection using artificial intelligence. It is currently in the phase of machine intelligence and cloud-based development.

## 2. Materials and Methods

**Figure 1:**
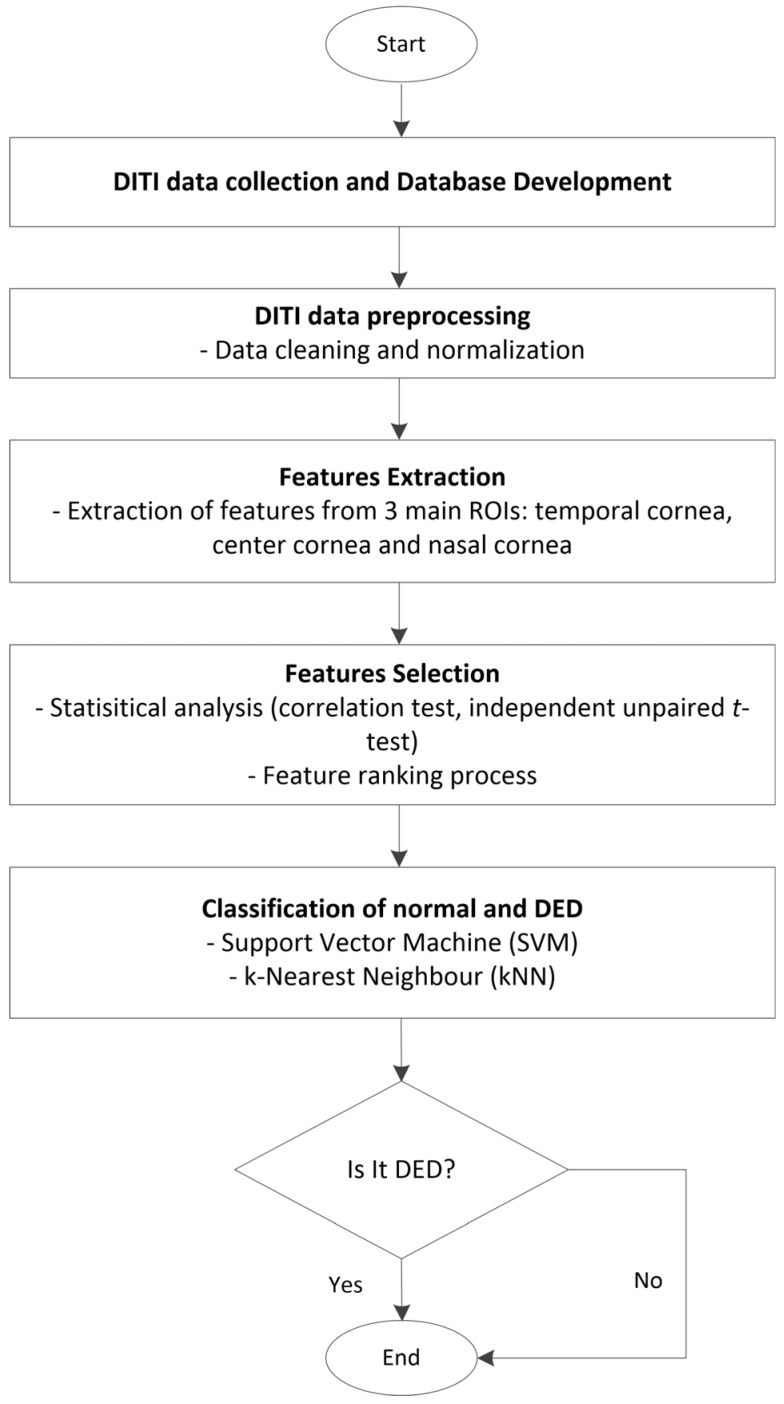
Overall workflow of the proposed DED screening system using DITI data.

The proposed methodology for screening DED is based on Digital Infrared Thermal Imaging (DITI). It involves several key steps: DITI data collection, database development, data preprocessing, feature extraction, feature selection, and classification. Each step is designed to ensure accurate identification of relevant thermal patterns associated with DED. Figure 1 illustrates the overall workflow of the proposed DED screening system using DITI data.

### 2.1. Study Design and Data Acquisition

This study was conducted in collaboration with the Optometry and Vision Science Programme at Universiti Kebangsaan Malaysia (UKM), Kuala Lumpur campus. Data collection was carried out at the Optometry Clinic through a structured effort involving optometrists and optometry students. The dataset comprises DITI focused on the ocular regions of both the right and left eyes. Prior to participation, informed consent was obtained, and participant anonymity was strictly maintained. A total of 40 subjects were randomly selected, comprising a diverse group of individuals regardless of age, gender, or ethnicity, resulting in 80 eyes (58 diagnosed with DED and 22 classified as normal). Participants were included based on their availability and willingness to participate, with clinical diagnosis (based on TBUT and clinical evaluation) used to determine group allocation. No age or gender matching was performed between the DED and control groups. To ensure data reliability and reduce potential confounding factors, the following exclusion criteria were applied: recent ocular surgery, current use of medications affecting tear film stability, ongoing DED treatments, pregnancy or breastfeeding, use of eye drops within 6 h prior to imaging, and contact lens wear within the past two weeks.

Clinical DED detection was performed using a dual-assessment approach that integrated both objective and subjective diagnostic tools, each validated by experienced optometrists. The TBUT test was employed to assess the stability of the tear film. A fluorescein dye was applied to the ocular surface using a moistened strip, and the time interval between the final blink and the appearance of the first dry spot on the pre-corneal tear film was measured under a cobalt blue filter using a slit lamp. A TBUT value of less than 5 s was considered indicative of tear film instability and thus, DED, in line with clinical standards. In addition to this objective measure, participants were asked to complete the OSDI questionnaire. This self-administered, 12-item tool is designed to assess the frequency and severity of DED symptoms such as burning, irritation, blurred vision, and visual discomfort over the preceding two to four weeks. The OSDI score was calculated using the standard formula [19], and scores exceeding 13 were interpreted as suggestive of DED. The formula is as follows,

(1) $$OSDI\;Score=\frac{\left(sum\;of\;scores\;for\;all\;questions\;answered\right)\times 100}{\left(total\;number\;of\;questions\;answered\right)\times 4}$$

The combination of both clinical signs and self-reported symptoms provided a comprehensive basis for participant diagnosis.

**Figure 2:**
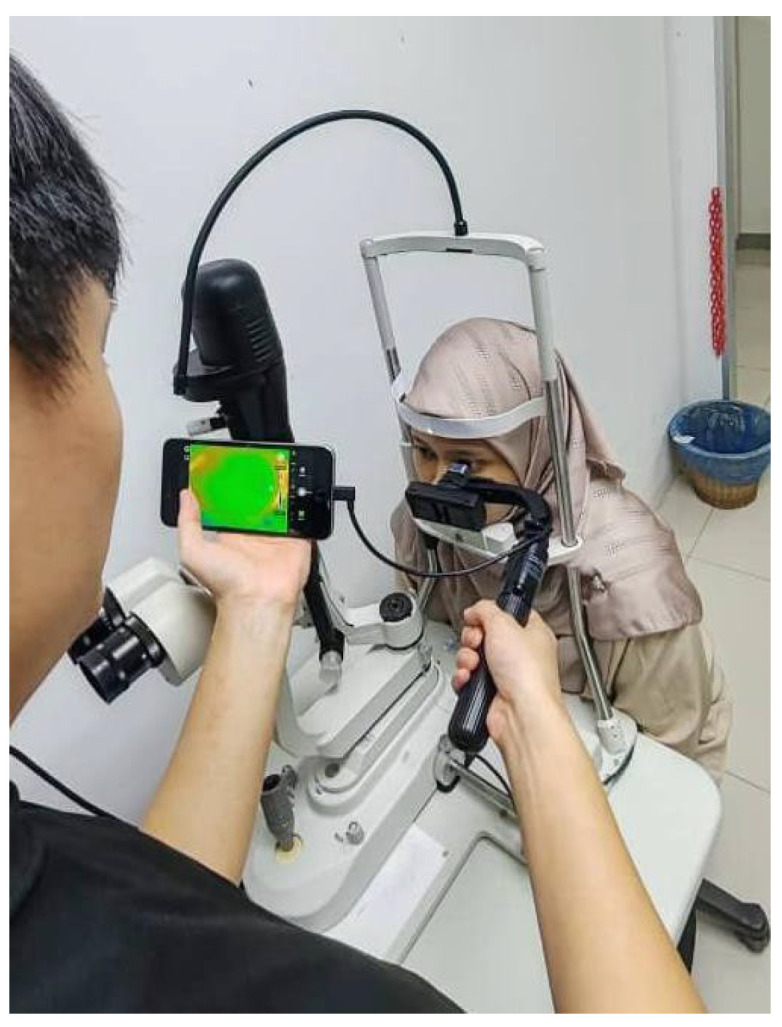
DITI data captured using InfiRay P2 Pro that was mounted on a selfie stick using a slit lamp chin rest.

Before conducting thermal imaging, participants were instructed to rest in the examination room for 10 min to allow their ocular surface temperature to stabilize. The room was maintained at a temperature of approximately 20–24 °C with humidity levels between 60–68%. Participants were asked to blink normally for a few seconds and then open their eyes as widely as possible. Immediately afterward, DITI images were captured over a continuous 5-s interval, with frames recorded every 0.3 s, during the time window of 8:00 AM to 4:30 PM. To ensure consistency in positioning and imaging distance, the IRT camera, specifically the InfiRay P2 Pro (iPhone version) by InfiSense Technology Co., Ltd. (Wuxi, China) that was mounted on a selfie stick while participants were stabilized using a slit lamp chin rest, as illustrated in Figure 2. The camera offered a thermal resolution of 256 × 192 pixels at 25 Hz, and images were captured using an iPhone 6s Plus running iOS 14.4.2. Capturing high-quality thermal images proved particularly challenging among subjects with more severe DED symptoms, as they often had difficulty keeping their eyes wide open and maintaining position for the entire duration. Consequently, repeated image captures were occasionally necessary to meet the predefined quality standards.

Temperature data were extracted using the ‘Thermal P2 Mobile’ application. Ocular Surface Temperature (OST) values were recorded at three predefined regions of interest (ROIs): nasal cornea (NC), temporal cornea (TC), and center cornea (CC), as described in Table 1. The extracted OST data were analyzed using JASP software (Version 0.18.3) to conduct preliminary statistical evaluations and explore potential correlations between temperature distribution and DED status. These thermal features were also used as inputs for classification models developed in MATLAB R2023b, enabling ML-based differentiation between DED and normal eyes. The integration of well-defined clinical assessments, strict measurement protocols, and controlled imaging conditions ensures the reliability and reproducibility of the dataset, forming a robust foundation for further statistical and predictive analyses.

### 2.2. Preprocessing of DITI Data

Preprocessing of the DITI data was a critical step to ensure data quality, balance, and suitability for both statistical analysis and ML applications. The initial dataset comprised 22 thermographic eye images from normal subjects and 58 from individuals diagnosed with DED, presenting a notable class imbalance. While such an imbalance can significantly affect ML model performance by biasing predictions toward the majority class, it may also impact statistical tests like the *t*-test, particularly if assumptions of equal variances and normal distribution are not met. To mitigate these challenges, a data cleaning and balancing process was implemented, resulting in a representative subset of 20 normal and 20 DED eye images. This process included the exclusion of extreme or outlier cases, specifically those with very severe DED, to prevent these atypical values from disproportionately influencing model training and statistical evaluations. The decision to exclude severe cases was made to enhance model generalizability for typical DED detection scenarios and to reduce potential bias introduced by extreme clinical presentations.

As part of the preprocessing step, the Shapiro-Wilk test was initially conducted to evaluate the general distribution of the dataset, guiding the decision to apply parametric methods in subsequent analysis. In this test, a *p*-value greater than 0.05 suggests that the data does not significantly deviate from a normal distribution, thereby supporting the assumption of normality required for parametric tests. Conversely, a *p*-value less than 0.05 would indicate a non-normal (i.e., non-parametric) distribution. Since most of the variables in the dataset yielded *p*-values above this threshold, it was concluded that the data approximated a normal distribution. Therefore, the independent (unpaired) *t*-test, a parametric statistical method, was selected as an appropriate tool for comparing the mean values across the two groups.

This preprocessing and statistical validation step was crucial in ensuring a fair and statistically sound comparison between the normal and DED classes. By balancing the sample sizes and confirming the distributional assumptions, the dataset became more suitable for both inferential statistical analysis and ML model development. Moreover, removing extreme or unrepresentative data points reduced potential bias and enhanced the robustness and generalizability of the resulting classification models. This systematic approach provided a reliable foundation for subsequent thermal feature analysis and the development of data-driven screening tools for DED.

### 2.3. Statistical Analysis

**Figure 3:**
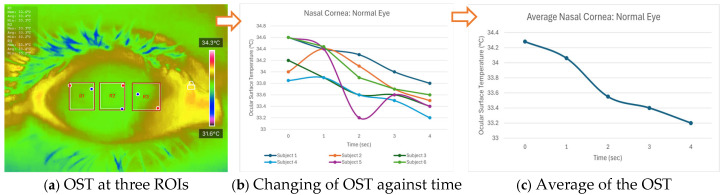
An example of how average OST was extracted for each ROI.

All processed DITI data, including OST measurements and participant diagnostic information, were documented in a standardized proforma and systematically compiled in Microsoft Excel for initial organization, tabulation, and visualization. Before applying statistical comparisons, the normality of specific thermal variables, including initial OST, cooling rate, and average OST across ROIs, was assessed using the Shapiro-Wilk test to confirm their suitability for parametric evaluation. Figure 3 shows an example of how the average OST was extracted for each ROI. When normality assumptions were satisfied, Analysis of Variance (ANOVA) and independent unpaired *t*-tests were applied to identify significant differences between the normal and DED eye groups.

Additional statistical analysis was conducted to investigate the potential associations between thermographic features and clinical diagnostic indicators. Pearson’s correlation coefficients were computed to evaluate the relationships between OST-based metrics such as initial temperature, average cooling rate, body temperature, and clinical measures, including OSDI scores and TBUT values. This analysis aimed to determine whether temperature-derived features could objectively reflect the severity or presence of DED symptoms. Given that the dataset was considered normally distributed, Pearson’s correlation was selected as the appropriate method due to its suitability for continuous, normally distributed variables [20]. The correlation coefficient (r) indicates the strength and direction of the linear association between variables, while the *p*-value assesses the statistical significance of the observed relationship.

### 2.4. Machine Learning Classification

To further validate the utility of DITI features in automated DED screening, a range of ML classification models were implemented using the Classifier Learner app in MATLAB R2023b. This environment enabled the comparison of multiple algorithms under consistent conditions, facilitating the identification of the most effective model for classifying eyes as either normal or DED-affected. Models evaluated included support vector machines (SVM) and k-nearest neighbours (k-NN) methods. Each model was trained using the same input features, primarily OST values from the three ROIs (NC, CC, and TC), and was evaluated using a cross-validation strategy to minimize overfitting and enhance model robustness.

Feature selection through ML ranking methods is essential for optimizing predictive models in DED screening using IRT. Key features identified include average OST, initial OST, and OST cooling rate. Average OST reflects the baseline thermal condition of the ocular surface, influenced by tear film stability and vascularization, where lower temperatures may indicate tear film deficiencies. Initial OST, measured immediately after eye opening, has been linked to ambient and body temperature, as well as tear film instability [21,22]. The OST cooling rate, representing the rate of temperature decline due to tear evaporation, has been widely validated as a biomarker for DED, with several previous studies by [15,23,24]. The OST cooling rate is calculated using the formula,

(2) $$Cooling\;Rate(\Delta {}^{\circ}C/second)=\frac{Temperature\_final-Temperature\_initial}{Elapsed\;Time(seconds)}$$

Despite international advancements, limited research in Malaysia highlights the importance of this study, considering local environmental factors such as climate and lifestyle, which can influence OST dynamics and DED prevalence.

To assess model performance, standard classification metrics including accuracy, sensitivity (recall), and specificity were derived from confusion matrices generated for each algorithm. These matrices, which detail the counts of true positives (TP), false positives (FP), true negatives (TN), and false negatives (FN), provided a comprehensive evaluation of each model’s ability to distinguish between DED and normal cases. Accuracy reflected the overall correctness of classifications, while sensitivity measured the model’s effectiveness in correctly identifying DED cases, and specificity assessed its ability to accurately recognize normal eyes. Furthermore, Receiver Operating Characteristic (ROC) curves were plotted to visualize classification performance, with the Area Under the Curve (AUC) serving as a key indicator of each model’s discriminative power between normal and DED conditions. This multi-metric evaluation approach ensured a balanced and robust assessment, which is crucial in medical diagnostics where minimizing false negatives is vital.

## 3. Results

### 3.1. DITI Dataset Processing and Analysis

For the statistical analysis, a balanced dataset comprising 40 eyes (20 normal and 20 diagnosed with DED was utilized. Table 2 summarizes the demographic and clinical characteristics of the participants. The mean age of the normal group was significantly higher (31.25 ± 17.57 years) compared to the DED group (25.63 ± 10.63 years, *p* < 0.001). This age difference arose because the study included subjects regardless of age, gender, or ethnicity. Interestingly, the DED group tended to be younger. Additionally, while both groups consisted predominantly of female participants, the proportion was notably higher in the DED group (85%) than in the normal group (65%), which aligns with established epidemiological patterns linking higher DED prevalence to females [25].

Clinically significant differences were observed between the two groups across key diagnostic parameters. TBUT, an indicator of tear film stability, was markedly lower in the DED group (2.37 ± 0.60 s) compared to the normal group (5.85 ± 1.14 s, *p* < 0.001), reflecting characteristic tear film instability in DED patients. Similarly, the OSDI scores were significantly elevated in the DED group (37.43 ± 6.83) relative to the normal cohort (15.90 ± 8.48, *p* = 0.035), indicating greater subjective symptoms of ocular discomfort.

While the difference in body temperature between the groups was statistically significant (DED: 33.77 ± 0.54 °C vs. Normal: 33.62 ± 0.92 °C, *p* < 0.001), the small absolute variation of approximately 0.15 °C may have limited clinical significance. This variance is likely attributable to measurement sensitivity or sample size effects rather than a true physiological distinction. Overall, these findings highlight clear demographic and clinical distinctions between normal and DED subjects, validating the group stratification employed in this study and reinforcing the dataset’s representativeness for subsequent analysis.

The analysis identified two primary thermal biomarkers for differentiating normal eyes from those affected by DED: the normalized average OST and the OST cooling rate across three regions of interest ROIs (NC, CC, and TC). These findings, summarized in Table 3, revealed consistent patterns in both temperature distribution and cooling behaviour that are indicative of ocular health status.

In terms of normalized average OST, the NC consistently exhibited the highest values in both groups, measuring 0.58 ± 0.20 in normal eyes and 0.51 ± 0.18 in DED eyes. This was followed by CC and TC, reflecting a stable temperature gradient across corneal regions regardless of disease presence. The relatively elevated temperature in the nasal region is likely due to its anatomical proximity to vascularized structures, contributing to localized heat retention.

More critically, the OST cooling rate demonstrated a clear discriminatory pattern between normal and DED eyes. Subjects with DED exhibited significantly higher (more negative) cooling rates across all ROIs, indicative of accelerated heat loss associated with tear film instability and increased evaporative stress, which are the key characteristics of DED pathology. The steepest cooling was observed in NC (–0.233 °C/s), followed by CC (–0.228 °C/s) and TC (–0.217 °C/s). In contrast, normal eyes showed much lower cooling rates, reflecting better tear film stability and ocular surface protection. In normal subjects, CC exhibited the highest cooling rate (–0.074 °C/s), with minimal differences compared to the NC (–0.071 °C/s) and TC (–0.074 °C/s) regions, underscoring effective thermal homeostasis in healthy eyes. Collectively, these findings highlight that reduced normalized average OST combined with elevated cooling rates are distinctive thermal signatures of DED, reinforcing their potential as reliable, non-invasive biomarkers for the classification and diagnosis of DED.

Table 4 presents the average OST values (mean ± SD) across three ROIs (NC, CC, and TC) measured at the starting point (0 s) and sequentially at 1, 2, 3, and 4 s. The Shapiro-Wilk test results indicate that the majority of temperature parameters follow a normal distribution, as reflected by *p*-values exceeding the 0.05 threshold. However, a few exceptions were observed, such as the starting OST in TC with a *p*-value of 0.035, suggesting minor deviations from normality in isolated cases.

Despite these few deviations, the overall dataset demonstrates strong adherence to parametric assumptions. Variance analysis further confirmed measurement stability, with standard deviations consistently low across both normal and DED groups, where none exceeding ±0.59. This consistent dispersion highlights the reliability of the thermographic measurements and supports the robustness of the data preprocessing and extraction methods.

Given these results, the use of parametric statistical tests, particularly the independent unpaired *t*-test, is appropriate for comparing OST values between normal and DED groups across different ROIs and time points. The general conformity to normal distribution and controlled variance ensures that subsequent statistical analyses can reliably detect meaningful physiological differences, minimizing the risk of inaccurate conclusions due to data irregularities or variability.

**Figure 4:**
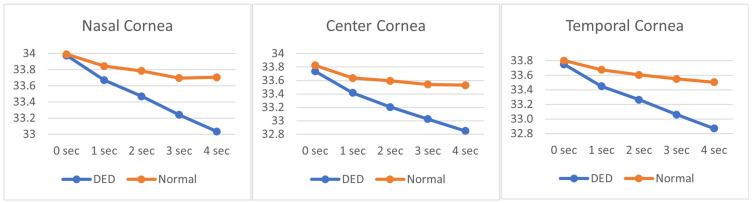
OST cooling dynamics over a 4-s interval across three distinct regions of interest (ROIs).

Figure 4 illustrates the OST cooling dynamics over a 4-s interval across three distinct ROIs. The temperature profiles range from approximately 32.8 °C to 34.0 °C, reflecting subtle but clinically relevant thermal variations. A clear distinction is observed between subjects with DED and those with normal ocular conditions. Across all ROIs, DED subjects consistently exhibit both a lower initial OST and a steeper cooling rate compared to normal subjects, as evidenced by the more pronounced downward slope of the DED curves. This accelerated cooling pattern in DED eyes may reflect compromised tear film stability and increased evaporative loss, characteristic of dry eye pathology.

Additionally, DITI assessments reveal a consistent temperature gradient across the corneal regions. The NC area demonstrates the highest average OST throughout the measurement period, with initial temperatures approximating 33.798 °C, followed by CC (33.520 °C) and TC (33.487 °C). This regional temperature variation may be indicative of differential tear film distribution, vascular proximity, or localized metabolic activity. Notably, the nasal region’s relative thermal preservation could be associated with its anatomical proximity to the medial canthus and lacrimal structures, potentially influencing tear dynamics and surface cooling behavior.

These findings highlight the potential of OST profiling as a non-invasive biomarker for distinguishing DED from normal ocular states, while also offering insights into the physiological thermoregulation patterns across different corneal zones.

### 3.2. Feature Selection and Correlation Analysis

**Figure 5:**
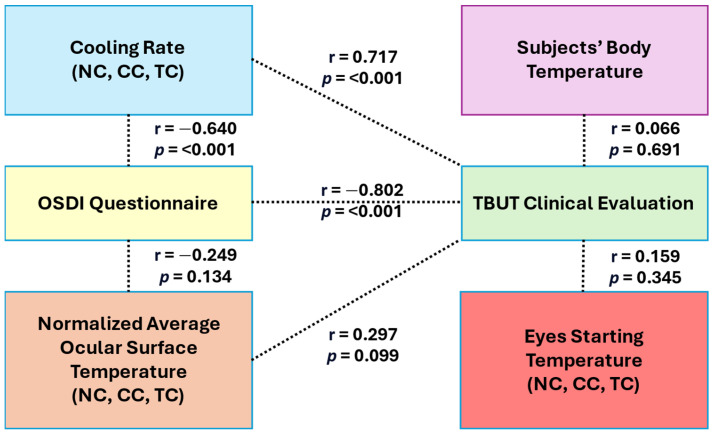
Pearson’s correlation test between clinical validation (TBUT and OSDI) with main features.

This section presents the selection of key features based on two primary criteria: statistical significance determined by independent unpaired *t*-tests (*p* < 0.05) and strong linear associations with clinical outcomes, as indicated by Pearson’s correlation coefficients (r > 0.5). Detailed statistical outcomes are summarized in Table 5, with inter-feature relationships visualized in Figure 5. Among all evaluated parameters, the OST cooling rate consistently demonstrated the strongest discriminatory power between normal and DED groups. Significant differences were observed across all ROIs (NC, CC, and TC) with highly significant *t*-values (*t* = −9.034 to −9.851, *p* < 0.001). Furthermore, the OST cooling rate for all ROIs showed strong positive correlations with clinical validation metrics, particularly TBUT (r = 0.717), reinforcing its role as a primary biomarker for DED classification.

In contrast, features such as normalized average OST, starting OST (0 s), and body temperature exhibited limited discriminatory capability. The normalized average OST did not show statistically significant differences between groups (*p* > 0.1 across all ROIs), although moderate positive correlations with TBUT were noted (e.g., r = 0.297, *p* = 0.099). Similarly, starting OST and body temperature yielded non-significant *p*-values (*p* = 0.345 and *p* = 0.691, respectively), indicating their limited utility as standalone diagnostic features due to weaker linear associations with clinical outcomes.

Secondary feature analysis revealed that OST measurements at later time points, particularly at 4 s, provided additional discriminatory value, with significant differences across all ROIs (*t* = −4.423 to −3.588, *p* < 0.001). NC exhibited the greatest separation between normal and DED groups at this time point. Additionally, the OSDI questionnaire maintained strong diagnostic relevance, showing a significant negative correlation with TBUT (r = −0.733, *p* < 0.001) and notable negative correlations with both OST cooling rate (r = −0.654, *p* < 0.001) and normalized average OST in selected ROIs. These correlations suggest that higher symptom severity aligns with faster cooling rates and reduced OST values. Based on these findings, the OST cooling rate was identified as the most robust and reliable feature for distinguishing DED, while secondary features such as OST at 4 s and OSDI scores further enhanced diagnostic accuracy. The integrated relationships between thermal imaging features, clinical evaluations, and subjective assessments are illustrated in Figure 5, underscoring the potential of combining DITI metrics with clinical tools for effective DED screening.

### 3.3. Evaluation of Classification Performance Using Machine Learning

#### 3.3.1. Support Vector Machine Classifier

For the SVM classifier, this study assessed five kernel functions: Linear, Quadratic, Cubic, Fine Gaussian, and Medium Gaussian using thermal imaging features from the DITI dataset to differentiate between normal and DED eyes. Feature selection was performed using ranked subsets of the Top-3, Top-5, and Top-10 most significant features to evaluate how feature quantity impacts model performance. As shown in Table 6, the Quadratic kernel demonstrated the most robust and balanced performance across these subsets, achieving the highest average metrics with an Accuracy of 90.54%, Sensitivity of 91.44%, Specificity of 88.20%, and the lowest Error rate of 9.46%. Notably, the Quadratic kernel maintained consistent performance regardless of whether fewer (Top-3) or more features (Top-10) were used, highlighting its stability and effectiveness in balancing DED detection while minimizing false positives.

The Linear kernel also showed strong performance, particularly excelling when using Top-5 features, where it achieved its highest accuracy (90.54%) and maintained the highest overall sensitivity (93.39%). This indicates that the Linear kernel is especially effective when focused on a concise, highly informative feature set, although its lower specificity (79.17%) suggests a greater tendency for false positives compared to the Quadratic kernel.

In contrast, the Cubic, Fine Gaussian, and Medium Gaussian kernels exhibited more variability depending on the number of features used. The Cubic kernel performed well with Top-10 features (90.54% accuracy), but its performance dropped significantly with fewer features, indicating sensitivity to feature selection. Both Gaussian kernels (Fine and Medium) showed declining accuracy and increasing error rates as more features were introduced, likely due to overfitting or sensitivity to noisy and less relevant variables.

Overall, this analysis highlights that careful selection of both the kernel function and the optimal number of features is crucial for maximizing classification performance. The Quadratic SVM kernel proved to be the most reliable across varying feature subsets, offering a strong balance of accuracy, sensitivity, and specificity for DED detection. The Linear kernel remains a valuable alternative when prioritizing sensitivity with a reduced feature set. Conversely, the inconsistent performance of the other kernels underscores the risks of overfitting and the importance of feature optimization in biomedical ML applications.

##### 3.3.2. k-Nearest Neighbours Classifier

The k-NN classification method in this study explored two critical parameters: the number of neighbours (k = 1, 3, 5) and the choice of distance metric (Euclidean, Chebyshev, and Mahalanobis). Using the top 10 selected features, model performance was evaluated based on accuracy (Acc), sensitivity (Sen), specificity (Spe), and error rate (Err), as detailed in Table 7. Among all configurations, the Euclidean distance metric consistently outperformed others, achieving the highest overall average performance (Acc = 88.74%, Sen = 89.73%, Spe = 86.65%, Err = 11.26%). The best individual result was recorded with k = 3, where Euclidean distance reached an impressive 91.89% accuracy, alongside balanced sensitivity and specificity, highlighting its strong generalization ability for DED classification.

While Euclidean distance emerged as the optimal choice, both Chebyshev and Mahalanobis distance metrics demonstrated stable and reliable performance, each averaging 86.04% accuracy across different k-values. The Chebyshev metric showed a slight advantage in specificity (85.52%), making it suitable for minimizing false positives, whereas Mahalanobis achieved marginally higher sensitivity (87.41%), indicating its effectiveness in correctly identifying DED cases.

Overall, these findings suggest that Euclidean distance with k = 3 provides the best balance between sensitivity and specificity for distinguishing normal and DED eyes within this dataset. Nevertheless, Chebyshev and Mahalanobis serve as viable alternatives, particularly in clinical contexts where prioritizing either sensitivity or specificity is essential for informed decision-making.

## 4. Discussion

**Figure 6:**
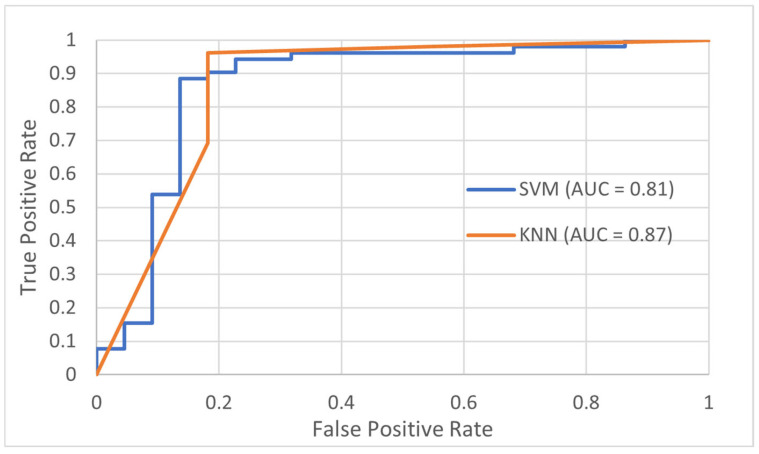
Linear SVM and Euclidean k-NN using top 3 features ROC results.

Table 8 presents a summary of the best-performing classifier methods in this study, highlighting k-NN and SVM approaches. The k-NN classifier, utilizing the Euclidean distance metric with k = 3 and the top 3 selected features, achieved the highest accuracy of 91.89%. Closely following, the SVM classifier with a Linear kernel and the top 10 features attained an accuracy of 91.80%. These results demonstrate that both classifiers are highly effective in distinguishing between normal and DED eyes, with k-NN showing a slight advantage while requiring fewer features, indicating its efficiency in handling reduced feature sets for this dataset. These optimized models formed the basis for further evaluation, including the ROC analysis illustrated in Figure 6.

This study highlights the potential of DITI as a non-invasive screening tool for DED by tracking temperature variations over time across NC, CC, and TC regions. Correlation analysis revealed a strong association between TBUT and OST cooling rate, where a lower cooling rate reflects greater tear film stability. As depicted in Figure 6, both k-NN and SVM classifiers achieved promising AUC values of 0.87 and 0.81, respectively. Additionally, independent *t*-tests confirmed significant differences in OST cooling rates between normal and DED groups (*p* < 0.001). These findings suggest that dynamic thermal metrics, particularly OST cooling rates, offer superior predictive capability compared to static measures like starting OST or body temperature, which demonstrated limited diagnostic value.

Based on clinical validation results, the cut-off values for TBUT and OSDI scores for normal eyes were TBUT ≥ 5 s and OSDI score < 13; for DED eyes, TBUT < 5 s and OSDI score > 13. From this statistical analysis study validated by optometrists, the cut-off values for normal eyes were TBUT ≥ 5.46 s and OSDI score < 15.078; for DED eyes, TBUT < 1.86 s and OSDI score > 42.51. The average OST measurements for three ROIs showed that NC had the highest average OST in normal eyes (33.804 °C) and DED eyes (33.488 °C), compared to CC and TC, with CC having the lowest average OST for DED (33.224 °C). This result has supported the previous finding where OST for DED is higher than normal, especially in the NC, CC, and TC regions [10]. For OST cooling rates, NC in DED eyes had the highest rate (−0.233 °C/s), followed by the CC (−0.228 °C/s) and TC (−0.217 °C/s). Since the nasal area had the highest starting OST, the OST cooling rate calculated also contributed to the high rate (slope is steeper in the graph of NC compared to CC and TC).

Previous studies [26,27] have reported that individuals with DED tend to exhibit lower starting OST and higher average OST cooling rates compared to normal subjects. The findings of this study further suggest a link between DED and increased tear evaporation. Additionally, research by [15] highlighted that patients with evaporative dry eye (EDE) typically present with unstable tear film (TBUT < 5 s) despite having a normal tear quantity (Schirmer test > 5 mm), leading to elevated tear evaporation rates, often indicative of meibomian gland dysfunction. In contrast, patients with aqueous-deficient dry eye (ADDE) generally maintain stable tear film (TBUT > 5 s) but suffer from reduced tear production (Schirmer test < 5 mm), resulting in low evaporation rates despite inadequate tear volume.

Besides, several studies have employed infrared thermography (IRT) cameras for screening and detecting DED. For instance, Ref. [28] developed a custom non-contact infrared (IR) thermal imaging system to measure ocular surface temperature variations, introducing two key parameters, which are temperature difference and compactness value, to differentiate between dry eye and normal eye groups. This system achieved 84% sensitivity, 83% specificity, and a 0.87 ROC area, demonstrating its potential as an effective non-contact diagnostic tool for dry eye detection. Similarly, ref. [29] presented a non-invasive method for automated dry eye detection using infrared thermography and Higher Order Spectra (HOS) for feature extraction. This system achieved 99.8% accuracy, sensitivity, and specificity for the left eye using PNN and KNN classifiers, and 99.8% accuracy, 99.9% sensitivity, and 99.4% specificity for the right eye using an SVM classifier. Additionally, ref. [30] developed an automated dry eye detection system using low-cost, low-quality infrared images with higher-order spectra (HOS) bispectrum features, achieving over 80% accuracy despite high noise levels. This method proves to be an efficient alternative to expensive, high-quality IR cameras. While previous studies have demonstrated high performance, the current study achieves comparable results and is among the first to use a smartphone-based handheld IRT camera, providing a more accessible, user-friendly, and cost-effective alternative to traditional high-end systems.

However, there were several challenges faced during this study. Firstly, different IRT instruments produce different DITI modalities for the proposed method, making it difficult to standardize results from other researchers who achieved high accuracy but used different features or parameters. For example, varying display resolutions can lead to different results during training with the same models in this study. Another challenge was that some individuals with DED were excluded from the study because they found it difficult to keep their eyes open for 5 s for the assessment, potentially leading to the loss of data in assessing DED prevalence in a population. There were also limitations in the manual temperature extraction method from the three ROIs through DITI, which was time-consuming at the data collection stage.

This study also identified five cases of very severe DED, in which subjects exhibit extreme clinical indicators, including TBUT values averaging 0.80 ± 0.45 s and OSDI scores of 36.67 ± 11.75, alongside a body temperature of 33.76 ± 0.54 °C. Although their OST cooling rates were lower than those observed in non-severe DED cases (indicating slower heat loss), they remained slightly higher than in normal eyes, reflecting persistent tear film dysfunction. Additionally, these subjects exhibited markedly elevated average OST values across all regions of interest (NC = 34.04 ± 0.75 °C, CC = 33.78 ± 0.85 °C, TC = 33.79 ± 0.79 °C), which remained sustained throughout the 0 to 4-s measurement period. This thermal profile is consistent with severe tear deficiency and pronounced tear film instability characteristic of advanced DED.

These severe cases were excluded to avoid skewing the classification model with potential outlier profiles, thereby enhancing robustness and ensuring optimal performance in the early screening of DED. In the future, the inclusion of severe DED cases could be further explored within a multi-class classification framework, enabling models to differentiate between varying degrees of disease severity (e.g., Normal, Mild, Moderate, Severe). Such an approach would enhance clinical applicability by facilitating both early detection and precise severity grading, thereby supporting comprehensive DED management strategies.

## 5. Conclusions

This proof-of-concept study explored the integration of smartphone-based infrared thermography (IRT) with machine learning techniques as a potential non-invasive framework for DED screening. While previous research has applied IRT in DED detection, this study is among the first to combine smartphone-enabled thermographic imaging with advanced classification algorithms.

The preliminary findings identified ocular surface temperature (OST) cooling rate as the most robust thermal biomarker for differentiating DED from normal eyes, supported by statistically significant group differences and strong correlations with clinical indicators such as TBUT. Among the evaluated machine learning models, the k-Nearest Neighbours (k-NN) classifier achieved the highest accuracy (91.89%), while the Quadratic Support Vector Machine (SVM) demonstrated stable performance (90.54%). These results indicate that DITI-derived features, when integrated with appropriate machine learning models, show potential for non-invasive, automated DED screening.

However, this study has certain limitations, including variability in IRT device specifications, manual thermal data extraction processes, and patient compliance during image acquisition. Future research should focus on expanding the dataset, utilizing higher-resolution thermographic sensors, and integrating complementary clinical assessments such as the Schirmer test to improve differentiation of DED subtypes. Additionally, developing models to grade DED severity could enhance clinical applicability and support personalized management strategies.

In conclusion, this study highlights that the integration of smartphone-based IRT with machine learning provides a practical, efficient, and scalable solution for early DED screening. This approach holds considerable promise for enhancing diagnostic accessibility and outcomes, particularly in resource-limited or remote healthcare settings.

## Figures and Tables

**Table 1:** Examples of DITI captured consecutively over 4 s for each subject, with ocular surface temperature recorded at each second. NC, CC, and TC denote Nasal Cornea, Central Cornea, and Temporal Cornea, respectively.

0 s	1 s	2 s	3 s	4 s
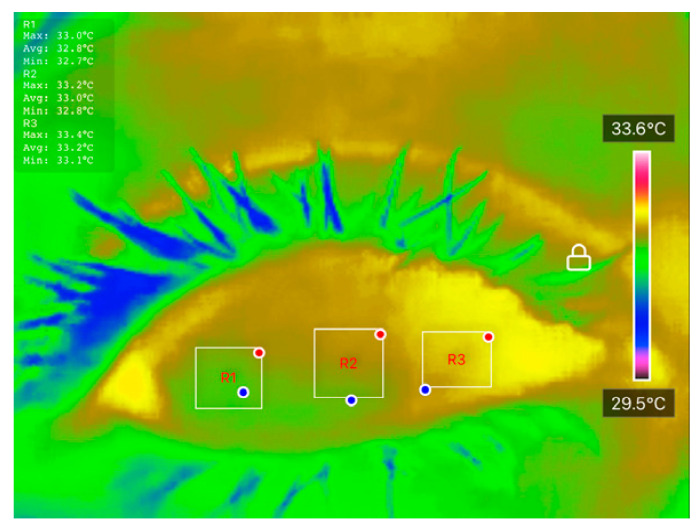	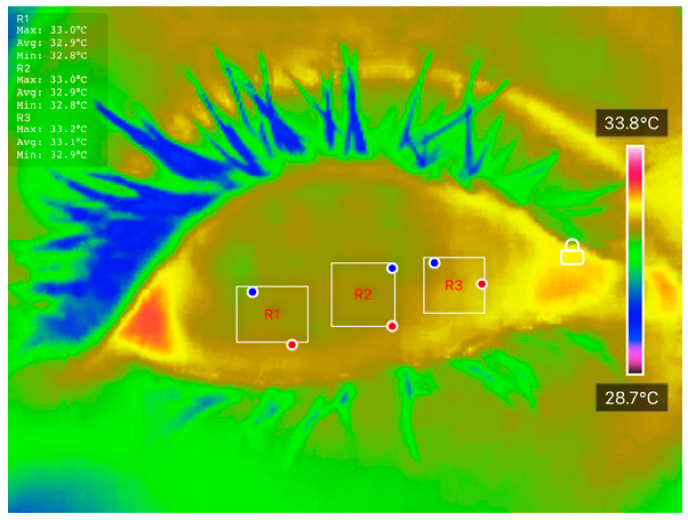	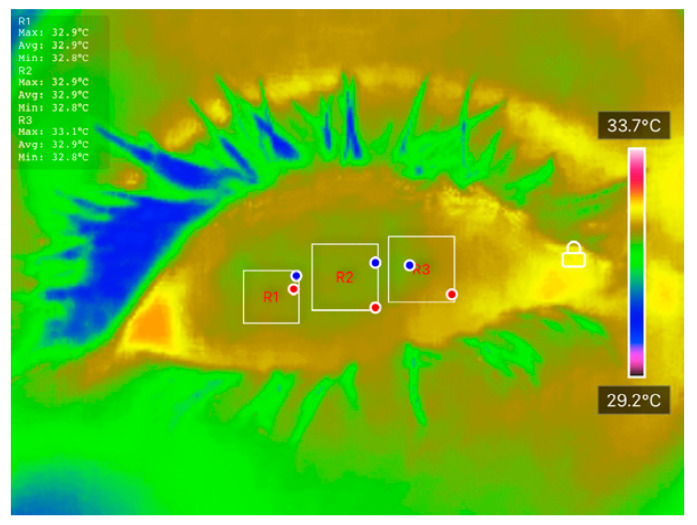	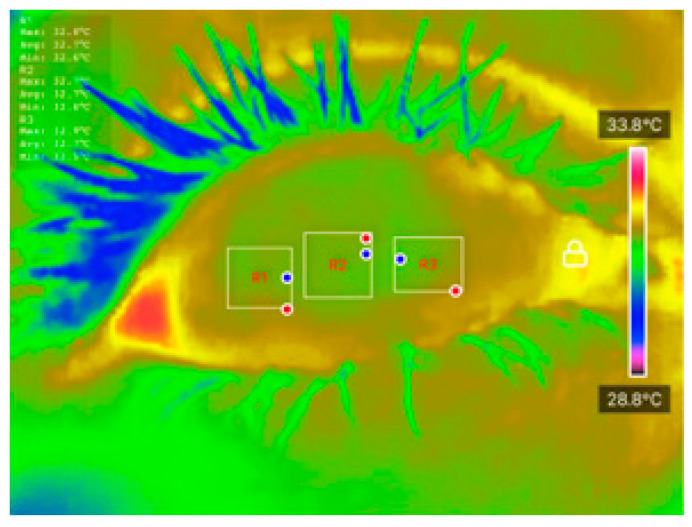	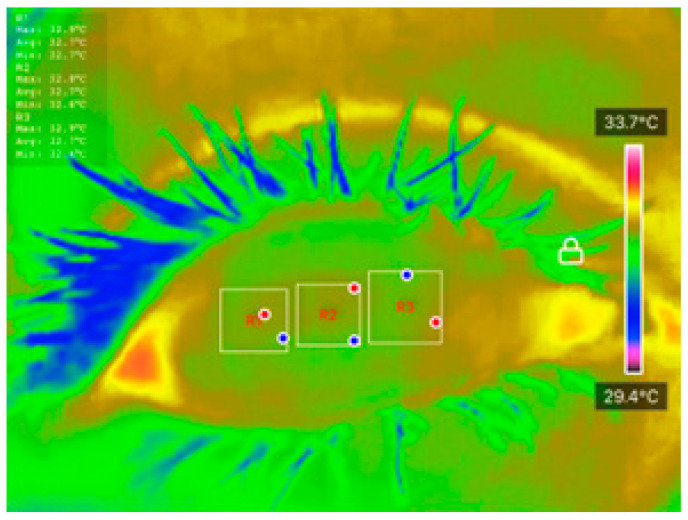
NC = 33.2 °C CC = 33.0 °C TC = 32.8 °C	NC = 33.1 °C CC = 32.9 °C TC = 32.9 °C	NC = 32.9 °C CC = 32.9 °C TC = 32.9 °C	NC = 32.7 °C CC = 32.7 °C TC = 32.7 °C	NC = 32.7 °C CC = 32.7 °C TC = 32.7 °C

**Table 2:** Summary of demographic and clinical characteristics of the participants.

Parameters	Normal	DED	Shapiro-*p*
Number of eyes	20	20	-
Age (year)	31.25 ± 17.57	25.63 ± 10.63	<0.001
Gender (female%, n)	65% (13)	85% (17)	-
TBUT (s)	5.85 ± 1.14	2.37 ± 0.60	<0.001
OSDI score	15.90 ± 8.48	37.43 ± 6.83	0.035
Body temperature (°C)	33.62 ± 0.92	33.77 ± 0.54	<0.001

**Table 3:** Normalized average OST and the OST cooling rate across three regions of interest (ROIs).

Parameter		Normal	DED
Normalized average OST (°C)	NC	0.58 ± 0.20	0.51 ± 0.18
	CC	0.62 ± 0.22	0.52 ± 0.21
	TC	0.57 ± 0.24	0.49 ± 0.22
Cooling rate of OST (°C/s)	NC	−0.071 ± 0.06	−0.233 ± 0.05
	CC	−0.074 ± 0.05	−0.228 ± 0.04
	TC	−0.074 ± 0.06	−0.217 ± 0.06

**Table 4:** Average OST values (mean ± SD) across three regions of interest (ROIs).

Parameter		Normal	DED	Shapiro-*p*
	NC	33.99 ± 0.44	33.99 ± 0.41	0.075
Starting OST 0 s (°C)	CC	33.83 ± 0.51	33.75 ± 0.45	0.515
	TC	33.80 ± 0.52	33.77 ± 0.41	0.035
	NC	33.85 ± 0.46	33.68 ± 0.42	0.342
OST at 1 s (°C)	CC	33.64 ± 0.50	33.42 ± 0.51	0.184
	TC	33.68 ± 0.51	33.47 ± 0.45	0.479
	NC	33.79 ± 0.53	33.46 ± 0.40	0.589
OST at 2 s (°C)	CC	33.60 ± 0.56	33.21 ± 0.46	0.539
	TC	33.61 ± 0.56	33.29 ± 0.45	0.230
	NC	33.70 ± 0.53	33.26 ± 0.36	0.769
OST at 3 s (°C)	CC	33.54 ± 0.57	33.02 ± 0.43	0.806
	TC	33.55 ± 0.55	33.08 ± 0.44	0.337
	NC	33.71 ± 0.51	33.06 ± 0.39	0.852
OST at 4 s (°C)	CC	33.53 ± 0.59	32.84 ± 0.45	0.253
	TC	33.51 ± 0.59	32.90 ± 0.46	0.133

**Table 5:** Results from independent unpaired *t*-tests on main features and secondary features.

Main Features		*t*	*p*
Normalized average OST (℃)	NC	−1.109	0.275
	CC	−1.483	0.147
	TC	−1.007	0.320
Starting OST (℃ at 0 s)	NC	−0.004	0.997
	CC	−0.504	0.617
	TC	−0.203	0.840
Cooling rate of OST (℃/s)	NC	−9.034	<0.001
	CC	−9.851	<0.001
	TC	−7.29	<0.001
**Secondary Features**		***t***	***p***
OST at 1 s (°C)	NC	−1.165	0.251
	CC	−1.361	0.182
	TC	−1.312	0.198
OST at 2 s (°C)	NC	−2.165	0.037
	CC	−2.359	0.024
	TC	−1.931	0.061
OST at 3 s (°C)	NC	−2.988	0.005
	CC	−3.241	0.003
	TC	−2.923	0.006
OST at 4 s (°C)	NC	−4.423	<0.001
	CC	−4.124	<0.001
	TC	−3.588	<0.001

**Table 6:** SVM assessment using the three top features. Acc, Sen, Spe, and Err represent accuracy, sensitivity, specificity, and error, respectively.

Kernel	Assessment	Top-3 Features	Top-5 Features	Top-10 Features	Average
Linear	Acc (%)	86.49	90.54	89.19	88.74
	Sen (%)	93.75	94.12	92.31	93.39
	Spe (%)	73.08	82.61	81.82	79.17
	Err (%)	13.51	9.46	10.81	11.26
Quadratic	Acc (%)	90.54	89.19	91.89	90.54
	Sen (%)	92.45	89.29	92.59	91.44
	Spe (%)	85.71	88.89	90.00	88.20
	Err (%)	9.46	10.81	8.11	9.46
Cubic	Acc (%)	86.49	77.03	90.54	84.68
	Sen (%)	90.38	85.71	92.45	89.52
	Spe (%)	77.27	60.00	85.71	74.33
	Err (%)	13.51	22.97	9.46	15.32
Fine Gaussian	Acc (%)	87.84	87.84	77.03	84.23
	Sen (%)	86.44	86.44	76.92	83.27
	Spe (%)	93.33	93.33	77.78	88.15
	Err (%)	12.16	12.16	22.97	15.77
Medium Gaussian	Acc (%)	85.14	81.08	78.38	81.53
	Sen (%)	83.61	79.69	77.27	80.19
	Spe (%)	92.31	90.00	87.50	89.94
	Err (%)	14.86	18.92	21.62	18.47

**Table 7:** k-NN assessment using the top three features.

Distance Technique	Assessment	k = 1	k = 3	k = 5	Average
Euclidean	Acc (%)	85.14	91.89	89.19	88.74
	Sen (%)	88.68	92.59	87.93	89.73
	Spe (%)	76.19	90.00	93.75	86.65
	Err (%)	14.86	8.11	10.81	11.26
Chebyshev	Acc (%)	85.14	86.49	86.49	86.04
	Sen (%)	88.68	86.21	85.00	86.63
	Spe (%)	76.19	87.50	92.86	85.52
	Err (%)	14.86	13.51	13.51	13.96
Mahalanobis	Acc (%)	82.43	87.84	87.84	86.04
	Sen (%)	86.79	87.72	87.72	87.41
	Spe (%)	71.43	88.24	88.24	82.63
	Err (%)	17.57	12.16	12.16	13.96

**Table 8:** Summary of the best classifier method results k.

Classifier Method	Parameter/Kernel Type	Features	Accuracy (%)
k-NN	Euclidean + (k = 3)	Top-3	91.89
SVM	Linear	Top-10	91.80

## Data Availability

The datasets presented in this article are not readily available because the data are part of an ongoing study.

## Abbreviations

The following abbreviations are used in this manuscript:

DED	Dry Eye Disease
DITI	Digital Infrared Thermal Imaging
DL	Deep Learning
ML	Machine Learning
OST	Ocular Surface Temperature
NC	Nasal Cornea
CC	Center Cornea
TC	Temporal Cornea
TBUT	Tear Break Up Time
OSDI	Ocular Surface Disease Index
SVM	Support Vector Machine
k-NN	k-Nearest Neighbours
